# The association between the urinary chromium and blood pressure: a population-based study

**DOI:** 10.1186/s12872-024-03918-8

**Published:** 2024-05-11

**Authors:** Dan Liang, Chang Liu, Mei Yang

**Affiliations:** 1 Department of Endocrine, The First People’s Hospital of Chongqing Liangjiang New Area, Chongqing, China; 2 School of Medicine, Nankai University, Tianjin, China

**Keywords:** Urinary chromium, Hypertension, Blood pressure, NHANES

## Abstract

**Background and Aim:**

The impact of trace elements and heavy metals on human health has attracted widespread attention. However, the correlation between urinary chromium concentrations and blood pressure remains unclear and inadequately reported, and the aim of this study was to investigate the relationship between urinary chromium concentrations and blood pressure in adults in the United States (US).

**Methods:**

We utilized data from the National Health and Nutrition Examination Survey (NHANES) 2017–2018 for this study. Multivariate logistic regression and multivariate linear regression were used to explore the association of urinary chromium concentrations with hypertension and blood pressure. Additionally, we also performed subgroup analysis and restricted cubic splines (RCS).

**Results:**

A total of 2958 participants were enrolled in this study. The overall mean systolic blood pressure and diastolic blood pressure were 123.98 ± 0.60, 72.66 ± 0.57 mmHg, respectively. The prevalence of hypertension was found in 41.31% of the whole participants. In the fully adjusted model, we did not observe a correlation between urinary chromium concentrations and the risk of hypertension and systolic blood pressure. However, we found a negative association between urinary chromium concentrations and diastolic blood pressure. In subgroup analysis, we observed a positive association between urinary chromium and the risk of hypertension among participants older than 60 years of age and those who were Non-Hispanic Black. The interaction term highlighted the influence of age and race on this positive association. We also found a negative association of urinary chromium with diastolic blood pressure in male, participants who were current smokers, overweight, and other races, as well as those without alcohol use and anti-hypertensive drug use. However, the interaction term only revealed the influence of alcohol consumption on the negative association.

**Conclusion:**

Our study suggested that urinary chromium concentrations may show a negative association with diastolic blood pressure and this association was significantly dependent on alcohol consumption. Besides, a positive association between urinary chromium and the risk of hypertension was also found among participants older than 60 years of age and those who were Non-Hispanic Black.

## Introduction

Chromium holds significance as a vital trace element for human health; nevertheless, the precise workings of its impact on the human body remain not entirely elucidated [1]. Simultaneously, chromium emerges as a potentially hazardous heavy metal, ranking among the prevalent environmental contaminants, notably prevalent in industrial settings such as tanneries [2]. The elevated chromium concentrations in water and soil, resulting from a spectrum of both natural and human-induced activities, have sparked considerable concern regarding the environmental ramifications of chromium pollution [3, 4].

The escalating prevalence of hypertension has elevated it to a pressing public health concern. Furthermore, hypertension stands as a pivotal risk factor for a spectrum of ailments, including coronary heart disease, heart failure, and stroke [5, 6]. A cross-sectional study has brought to light an independent association between serum chromium levels and hypertension [7]. Lower plasma chromium levels have been linked to hyperglycemia, hyperinsulinemia, hypertension, and insulin resistance [8]. Intriguingly, a meta-analysis contradicts these findings by revealing that chromium supplementation significantly diminishes both systolic and diastolic blood pressure [9]. Conversely, another meta-analysis indicates that chromium supplementation doesn't notably alter systolic blood pressure, with no discernible correlation between blood pressure levels and the dose or duration of chromium supplementation [10].

The relationship between long-term chromium exposure and hypertension is contradictory. A study focused on preschool children proposed a negative association between chromium and barium exposure and both blood pressure and hypertension [11]. However, another study indicated that residing in regions with heightened chromium and arsenic exposure was correlated with an increased risk of hypertension [12]. Currently, the association between chromium concentrations and blood pressure in the noninstitutionalized general population in the US has yet to be explored. In this study, we attempted to investigate the relationships between urinary chromium and blood pressure, in a nationally representative sample of US adults.

## Materials and methods

### Study population

We sourced our data from NHANES, a cross-sectional study with the objective of evaluating the health and nutrition status of the U.S. population, administered by the National Center for Health Statistics (NCHS) of the U.S. Center for Disease Control and Prevention (CDC). All NHANES data were publicly available at https://www.cdc.gov/nchs/nhanes/. The NHANES survey was a national research program conducted in a 2-year repeated cycle with continuously updated survey data. The NHANES study design used a complex stratified, multistage probability sampling method to assess the health and nutrition status of the U.S. population, ensuring a degree of representativeness in participant recruitment. The Research Ethics Review Committee of NCHS approved all NHANES study protocols and obtained written informed consent from all survey participants or parents and/or legal guardians of participants under 16 years of age.

**Fig. 1:**
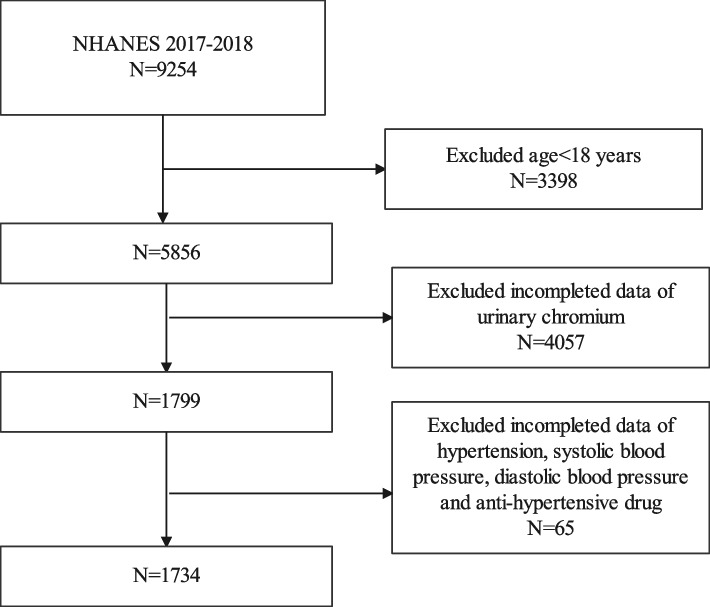
Flowchart of the sample selection from the 2017–2018 National Health and Nutrition Examination Survey (NHANES)

Our study used data from NHANES 2017–2018, as this is the only survey cycle that included both urinary chromium and blood pressure data. Our study initially included 9254 participants and after excluding participants younger than 18 years (*n* = 3398), lack of data on urine chromium (*n* = 4057) and lack of data on hypertension, systolic blood pressure, diastolic blood pressure, and use of anti-hypertensive medication (*n* = 65), 1734 participants were eventually included in our final analysis (Fig. 1).

## Exposure and outcome definitions

The urinary chromium concentrations were designed as an exposure variable. The reason for choosing urine chromium as an exposure variable is that urinary chromium levels can reflect not only recent exposure but also the cumulative effects of long-term chromium intake. The chromium, upon absorption, tends to accumulate in various tissues over time. This stored chromium is gradually released into circulation and eventually excreted in urine [13, 14]. The half-life of chromium in serum is 40 months, while the half-life of chromium in urine is 129 months [15, 16]. The random (or spot) urine samples were collected from the participants after confirming the absence of background contamination in the collection material. Measurement of urinary chromium levels was conducted using inductively coupled plasma mass spectrometry (ICP-MS), a multi-element analytical technique capable of incorporating dynamic reaction cell techniques (DRC) for trace element analysis. This method achieved rapid and accurate quantification of urinary chromium. Urine samples were processed, stored, and shipped to the Division of Laboratory Sciences, National Center for Environmental Health, Centers for Disease Control and Prevention, Atlanta, GA for analysis. Upon receipt, urine samples were stored at ≤ -20 °C until they were dispatched to the National Center for Environmental Health for testing.

Hypertension, systolic, and diastolic blood pressure were designed as outcome variables. After 5 min of quiet rest and after determining the participant's maximum inflation level (MIL), three consecutive blood pressure (BP) readings were obtained. If BP measurements were interrupted or incomplete, a fourth attempt could be made. All BP measurements (systolic and diastolic) were performed at the Mobile Examination Center (MEC).

Exclusion criteria for participants encompassed specific conditions on both arms, such as rash, gauze dressing, plaster, edema, paralysis, tubal, open ulcer or wound, arm blight, arteriovenous shunt, and mastectomy. BP measurements predominantly took place in the right arm unless particular conditions prohibited its use or the participant cited reasons against measuring in the right arm. Each participant underwent 1–4 BP readings during the study, with those lacking any BP readings being excluded. If they had only one BP reading, then this was the final record. For participants with multiple BP readings, the initial reading was consistently excluded, and the BP record represented the average of subsequent readings. Hypertension was defined based on a self-reported diagnosis of hypertension, diastolic blood pressure ≥ 90 mmHg or systolic blood pressure ≥ 140 mmHg, or the use of antihypertensive medications [17].

## Covariates

Covariates included in this study encompassed age, sex, race, education level, the ratio of family income to poverty (PIR), body mass index (BMI), serum calcium, serum phosphorus, serum creatinine, serum uric acid, urine albumin to creatinine ratio (uACR), estimated glomerular filtration rate (eGFR), total cholesterol, urinary lead, urinary cadmium, alcohol consumption, smoking status, diabetes mellitus (DM), and use of anti-hypertensive medications. BMI was classified as < 25, 25–29.9, and ≥ 30 kg/m2, which corresponded to normal weight, overweight, and obese population for all participants. DM was defined based on a self-reported diagnosis of diabetes mellitus, 2-h plasma glucose ≥ 200 mg/dL in an oral glucose tolerance test, HbAlc ≥ 6.5%, use of oral hypoglycemic agents, or fasting glucose ≥ 126 mg/dL [18]. All detailed measurement processes of study variables were publicly available at www.cdc.gov/nchs/nhanes/.

## Statistical analysis

All analysis was performed using R version 4.2.1 (http://www.R-project.org, The R Foundation). All statistical analysis was conducted in accordance with CDC guidelines and appropriate NHANES sampling weights were applied, which illustrated the complex multi-stage cluster survey design in the analysis. Continuous variables were presented as mean with standard deviation and categorical variables were presented as percentages. The comparison between groups based on urinary chromium concentrations (tertiles) employed a weighted Student's t-test for continuous variables and a weighted chi-square test for categorical variables. Multivariate logistic regression models were used to explore the independent relationship between urinary chromium and the risk of hypertension in three different models. Multivariable linear regression models were also performed to explore the independent association of urinary chromium with systolic blood pressure and diastolic blood pressure after adjusting for potential confounding factors. No covariates were adjusted for in Model 1, and in Model 2, age, sex, and race were adjusted. In Model 3, adjustments were made for age, sex, race, education levels, PIR, BMI, serum phosphorus, serum creatinine, serum uric acid, eGFR, uACR, total cholesterol, urinary lead, urinary cadmium, alcohol consumption, smoking status, DM, and use of anti-hypertensive medications. Subgroup analyses stratified by age (< 60/ >  = 60), sex (male/female), race (Mexican American/Non-Hispanic White/Non-Hispanic Black/Other races), BMI (normal weight/overweight/obesity), diabetes (yes/no), alcohol consumption (yes/no), smoking status (never/former/now), and use of anti-hypertensive medications (yes/no) were also performed by stratified multiple regression analysis. In addition, an interaction term was added to test the heterogeneity of associations between the subgroups. To further investigate the association between urinary chromium concentrations and diastolic blood pressure, restricted cubic splines (RCS) with 3 knots at the 10th, 50th, and 90th percentiles were performed.

## Results

### Baseline characteristics of the enrolled participants

Table 1 illustrates the population characteristics and covariates, presenting the weighted distribution of included participants based on urinary chromium tertiles. A total of 1734 subjects, with a mean age of 47.64 ± 0.99 years were recruited in our study, of which 48.82% were males and 51.18% were females. The ranges of urinary chromium concentrations tertiles were <  = 0.13, 0.13–0.22. 0.22–41.79ug/L, respectively. The overall mean systolic blood pressure and diastolic blood pressure were 123.98 ± 0.60, and 72.66 ± 0.57 mmHg, respectively. The prevalence of hypertension was found in 41.31% of the whole participants. Among different urinary chromium concentrations tertiles, we found significant differences in age, serum creatine, eGFR, serum phosphorus, systolic blood pressure, diastolic blood pressure, and DM. No significant difference was observed in serum calcium, total cholesterol, serum uric acid, uACR, sex, race, PIR, education levels, BMI, alcohol consumption, smoking status, and hypertension. Participants in the highest tertile exhibited an older age, a higher likelihood of diabetes, higher urinary lead and urinary cadmium, elevated serum creatinine, and serum phosphorus, increased systolic blood pressure, decreased eGFR, and lower diastolic blood pressure compared to those in the lowest tertile of urinary chromium concentrations.

**Table 1:** Baseline characteristics of the study population

Urinary chromium	All participants	Q1 (< = 0.13)	Q2 (0.13–0.22)	Q3 (0.22–41.79)	*P* value
Age (year)	47.64 (0.99)	45.91 (1.16)	47.43 (1.59)	51.22 (1.16)	**0.004**
Serum calcium (mmol/L)	2.33 (0.01)	2.33 (0.01)	2.33 (0.01)	2.33 (0.01)	0.96
Serum creatinine (mg/dl)	0.88 (0.01)	0.86 (0.01)	0.85 (0.03)	0.93 (0.02)	**0.04**
eGFR (ml/ min/l.73 m^2)	94.55 (1.05)	96.36 (1.33)	96.45 (1.34)	90.37 (1.49)	**0.003**
Total cholesterol (mmol/L)	4.83 (0.05)	4.89 (0.07)	4.86 (0.12)	4.72 (0.06)	0.1
Serum uric acid (umol/L)	320.52 (3.21)	316.86 (5.16)	316.81 (9.96)	328.90 (4.30)	0.28
Serum phosphorus (mmol/L)	1.15 (0.01)	1.14 (0.01)	1.13 (0.01)	1.18 (0.01)	**0.01**
uACR (mg/g)	41.51 (6.35)	34.48 (6.30)	53.05 (32.09)	52.93 (19.01)	0.63
Urinary lead (ug/L)	0.42 (0.02)	0.33 (0.02)	0.57 (0.02)	0.59 (0.09)	** < 0.0001**
Urinary cadmium (ug/L)	0.28 (0.01)	0.22 (0.01)	0.36 (0.05)	0.57 (0.02)	** < 0.0001**
Systolic blood pressure (mmHg)	123.98 (0.60)	122.85 (0.74)	126.69 (2.76)	125.60 (0.64)	**0.04**
Diastolic blood pressure (mmHg)	72.66 (0.57)	72.88 (0.70)	75.53 (1.41)	71.48 (0.66)	**0.02**
Sex (%)					0.75
Female	51.18 (0.02)	52.07 (3.00)	52.20 (5.69)	49.10 (3.52)	
Male	48.82 (0.02)	47.93 (3.00)	47.80 (5.69)	50.90 (3.52)	
Races (%)					0.73
Mexican American	9.92 (0.02)	10.74 (2.05)	9.89 (2.94)	8.26 (2.42)	
Non-Hispanic Black	11.18 (0.01)	9.40 (1.39)	11.95 (2.34)	14.63 (1.88)	
Non-Hispanic White	61.53 (0.04)	60.39 (3.43)	62.97 (7.58)	63.51 (3.07)	
Others	17.36 (0.02)	19.48 (2.13)	15.19 (5.67)	13.60 (1.89)	
PIR (%)					0.3
< 1	12.03 (0.01)	13.25 (1.68)	17.92 (5.72)	13.19 (1.69)	
1–4	43.38 (0.03)	47.54 (2.85)	58.99 (8.37)	49.43 (4.02)	
> 4	33.22 (0.03)	39.21 (3.20)	23.08 (5.71)	37.37 (5.03)	
Educational levels (%)					0.73
Less than 9th grade	3.45 (0.01)	3.30 (0.61)	3.53 (1.49)	3.73 (0.78)	
9-11th grade	8.29 (0.01)	6.93 (1.23)	12.78 (2.50)	9.91 (1.42)	
High school graduate	31.48 (0.02)	31.56 (2.82)	30.81 (7.46)	31.49 (3.79)	
Some college or AA degree	28.53 (0.02)	28.95 (2.19)	26.61 (4.80)	28.17 (3.18)	
College graduate or above	28.25 (0.03)	29.25 (3.48)	26.26 (5.82)	26.70 (4.56)	
BMI (%)					0.19
Normal weight	25.06 (0.02)	26.18 (2.73)	17.48 (5.91)	25.31 (2.46)	
Overweight	29.71 (0.02)	32.05 (2.17)	20.66 (5.35)	27.96 (2.54)	
Obesity	44.49 (0.02)	41.77 (2.81)	61.86 (10.42)	46.73 (2.82)	
Smoke (%)					0.39
Never	58.36 (0.03)	57.43 (3.38)	62.31 (3.99)	59.23 (2.84)	
Former	26.11 (0.02)	25.78 (3.10)	29.26 (3.80)	25.98 (3.40)	
Now	15.54 (0.02)	16.79 (1.72)	8.44 (2.77)	14.80 (1.37)	
Alcohol user (%)					0.64
Yes	79.84 (0.03)	80.46 (1.42)	81.46 (4.64)	78.16 (2.03)	
No	20.16 (0.01)	19.54 (1.42)	18.54 (4.64)	21.84 (2.03)	
DM (%)	15.10 (0.01)	12.56 (1.54)	26.88 (5.89)	17.27 (2.15)	**0.01**
Anti-hypertensive drug (%)	4.53 (0.01)	4.65 (0.90)	2.15 (0.69)	4.90 (1.29)	0.2
Hypertension (%)	41.31 (0.03)	38.21 (3.81)	49.74 (7.83)	45.49 (3.63)	0.19

*eGFR* Estimated glomerular filtration rate, *uACR* Urine albumin to creatinine ratio, *PIR* Ratio of family income to poverty, *BMI* Body mass index

### The association between urinary chromium concentrations and hypertension

For unadjusted analyses (Model 1), urinary chromium concentrations were not associated with the risk of hypertension (OR: 1.20, 95%CI: 0.80–1.81, *p* = 0.35). After adjusting for potential confounders (Model 2 and Model 3), urinary chromium remained unassociated with the risk of hypertension (Model 2: OR: 1.03, 95%CI: 0.91–1.17, *p* = 0.56. Model 3: OR: 1.09, 95%CI: 0.94–1.26, *p* = 0.23).

To further assess the association between urinary chromium concentration and the risk of hypertension, we converted urinary chromium concentration from a continuous variable to a categorical variable (tertiles). The analysis revealed no significant correlation between urinary chromium concentration and the risk of hypertension. The unadjusted OR of the risk of hypertension for tertile 22 vs tertile 1 was 1.39 (95%CI: 0.866–2.52, *p* = 0.85). The fully adjusted OR of the risk of hypertension for tertile 3 vs tertile 1 was 1.08 (95%CI: 0.46–2.25, *p* = 0.17) (Table 2).

**Table 2:** Multivariate logistic regression models of hypertension with Urinary chromium

Hypertension	OR (95%CI)		
	Model 1	Model 2	Model 3
Continuous	1.20 (0.80, 1.81), *p* = 0.35	1.03 (0.91, 1.17), *p* = 0.56	1.09 (0.94, 1.26), *p* = 0.23
Categories			
Tertile 1	Reference	Reference	Reference
Tertile 2	1.60 (0.74, 3.44), *p* = 0.21	1.61 (0.59, 4.41), *p* = 0.31	1.39 (0.86, 2.52), *p* = 0.85
Tertile 3	1.35 (0.89, 2.06), *p* = 0.15	0.96 (0.58, 1.60), *p* = 0.87	1.08 (0.46, 2.25), *p* = 0.17

Model 1: No covariates were adjusted

Model 2: Age, sex and race were adjusted

Model 3:Age, sex, race, education levels, RIP, BMI, serum phosphorus, serum creatinine, serum uric acid, eGFR, uACR, total cholesterol, urinary lead, urinary cadimum, alcohol consumption, smoking status, DM, and use of anti-hypertensive medications were adjusted

### The association between urinary chromium concentrations and systolic blood pressure

No correlation was observed between urinary chromium concentration and systolic blood pressure in the unadjusted Model 1 and in Model 2, which was adjusted for age, sex, and race. In the fully adjusted Model 3, we found that each unit increase in urinary chromium concentration was associated with a 0.05 mmHg decrease in systolic blood pressure, although this difference was not statistically significant.

When we converted urinary chromium concentration from a continuous variable to a categorical variable, in the unadjusted model (Model 1), we found that the β (95% CI) of the systolic blood pressure for tertile 3 vs tertile 1 was 2.75 (95% CI: 0.63–4.86). In the fully adjusted model (Model 3), participants in the highest tertile exhibited a 1.54 mmHg increase in systolic blood pressure compared with those in the lowest tertile of urinary chromium concentration, although this difference did not reach statistical significance (Table 3).

**Table 3:** The association between systolic blood pressure and urinary chromium

Systolic blood pressure	β (95%CI)		
	Model 1	Model 2	Model 3
Continuous	0.5 (-0.44, 1.44), *p* = 0.27	-0.24 (-0.91, 0.42), *p* = 0.43	-0.05 (-0.64, 0.54), *p* = 0.88
Categories			
Tertile 1	Reference	Reference	Reference
Tertile 2	3.84 (-2.07, 9.75), *p* = 0.18	3.04 (-2.81, 8.88), *p* = 0.27	2.19 (-3.60, 7.97), *p* = 0.43
Tertile 3	2.75 (0.63, 4.86), ***p = 0.01***	-0.01 (-2.06, 2.03), *p* = 0.98	1.54 (-0.34, 3.42), *p* = 0.10

Model 1: No covariates were adjusted

Model 2: Age, gender, and race were adjusted

Model 3:Age, sex, race, education levels, RIP, BMI, serum phosphorus, serum creatinine, serum uric acid, eGFR, uACR, total cholesterol, urinary lead, urinary cadimum, alcohol consumption, smoking status, DM, and use of anti-hypertensive medications were adjusted

### The association between urinary chromium concentrations and diastolic blood pressure

For unadjusted analyses (Model 1), urinary chromium concentrations were not associated with diastolic blood pressure. In Model 2, we observed a negative association between urinary chromium concentrations and diastolic blood pressure (β:-0.61, 95%CI:-1.16 ~ -0.06, p = 0.03). This inverse association persisted in the fully adjusted model (Model 3), where urinary chromium concentrations were associated with a decrease in diastolic blood pressure (β:-0.53, 95%CI:-0.93 ~ -0.14, *p* = 0.01).

We converted urine chromium concentrations from continuous variable to categorical variables, and in the fully adjusted model (Model 3), we observed a decrease in diastolic blood pressure of 1.20 mmHg with each unit increase in urine chromium concentrations, though this difference was not statistically significant (Table 4).

**Table 4:** The association between diastolic blood pressure and urinary chromium

Diastolic blood pressure	β (95%CI)		
	Model 1	Model 2	Model 3
Continuous	-0.49 (-1.07, 0.10), *p* = 0.10	-0.61 (-1.16, -0.06), ***p = 0.03***	-0.53 ( -0.93, -0.14), ***p = 0.01***
Categories			
Quartile 1	Reference	Reference	Reference
Quartile 2	2.64 (-0.44, 5.73), *p* = 0.09	2.58 (-0.66, 5.81), *p* = 0.10	2.41 (-0.63, 5.44), *p* = 0.11
Quartile 3	-1.4 (-3.32, 0.52), *p* = 0.14	-1.79 (-3.93, 0.35), *p* = 0.09	-1.20 (-2.86, 0.46), *p* = 0.15

Model 1: No covariates were adjusted

Model 2: Age, gender, and race were adjusted

Model 3:Age, sex, race, education levels, RIP, BMI, serum phosphorus, serum creatinine, serum uric acid, eGFR, uACR, total cholesterol, urinary lead, urinary cadimum, alcohol consumption, smoking status, DM, and use of anti-hypertensive medications were adjusted

**Fig. 2:**
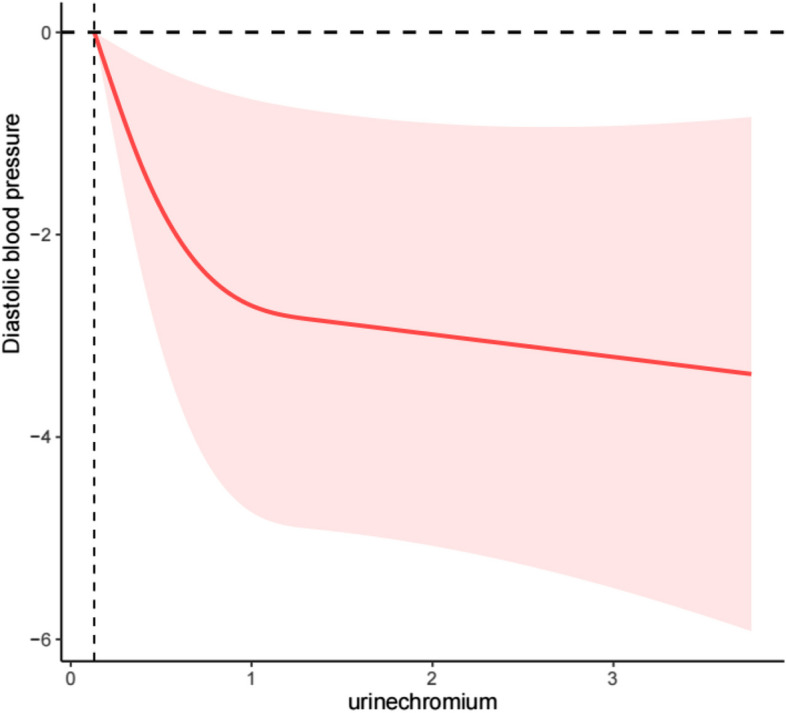
Restricted cubic spline (RCS) plot of the association between urinary chromuim concentrations and diastolic blood pressure. RSC plot of the association between urinary chromium concentrations and diastolic blood pressure (P nonlinear = 0.026)

To further explore the correlation between urinary chromium concentrations and diastolic blood pressure, we performed an RCS analysis to explore whether there was a nonlinear relationship between the two. Our results showed a J-shaped nonlinear correlation between urine chromium concentrations and diastolic blood pressure (P nonlinear = 0.026), indicating a decreasing trend in diastolic blood pressure with increasing urine chromium concentrations (Fig. 2).

## Subgroup analysis

**Fig. 3:**
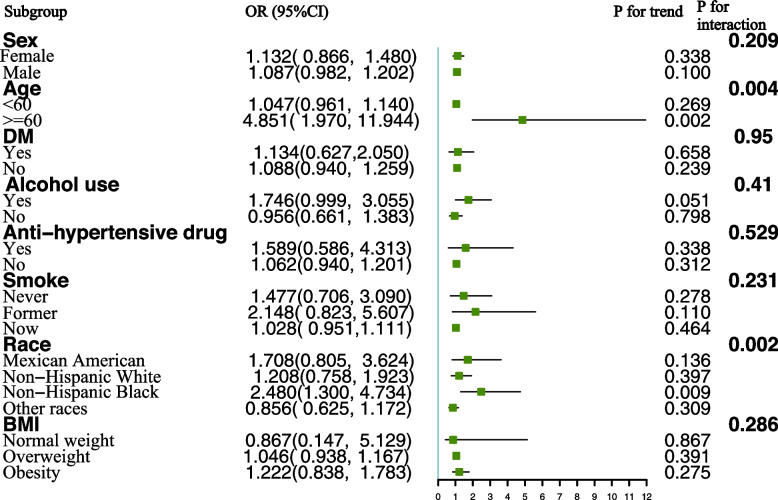
Subgropup analysis for the association between urinary chromium and Hypertension

**Fig. 4:**
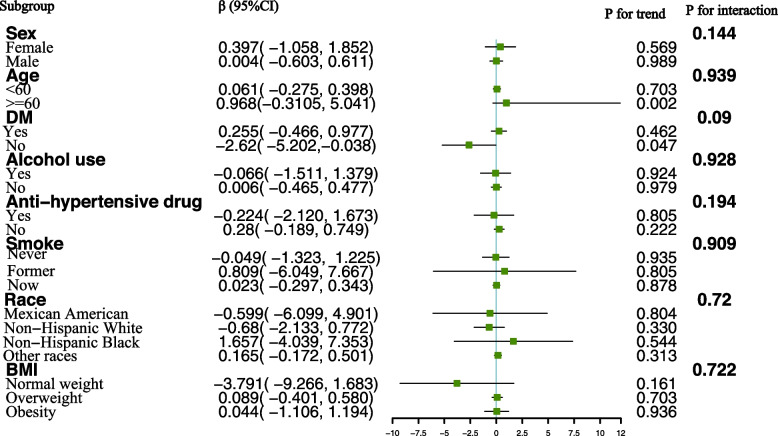
Subgroup analysis for the association between urinary chromium and systolic blood pressure

To delve deeper into the factors influencing the relationship between urinary chromium concentrations and hypertension, as well as systolic and diastolic blood pressure, a stratified analysis was conducted based on sex, age, race, BMI, diabetes status, alcohol consumption, smoking status, and use of anti-hypertensive medications. For the correlation between urinary chromium concentrations and hypertension, we observed a positive association among participants older than 60 years of age. An increase in urinary chromium concentration per unit correlated with a substantial 485.1% increased risk of hypertension in participants older than 60 years compared with their younger counterparts. A positive association between urinary chromium and the risk of hypertension was also found in subjects who were Non-Hispanic Black. The interaction term highlighted the influence of age (P for interaction = 0.004) and race (P for interaction = 0.002) on the association between urinary chromium concentrations and hypertension (Fig. 3). In contrast, no significant differences were suggested by the interaction test in the association between urinary chromium concentrations and systolic blood pressure across various stratifications. This indicates that there was no substantial dependence on stratified factors in the association between urinary chromium concentrations and systolic blood pressure (Fig. 4).

**Fig. 5:**
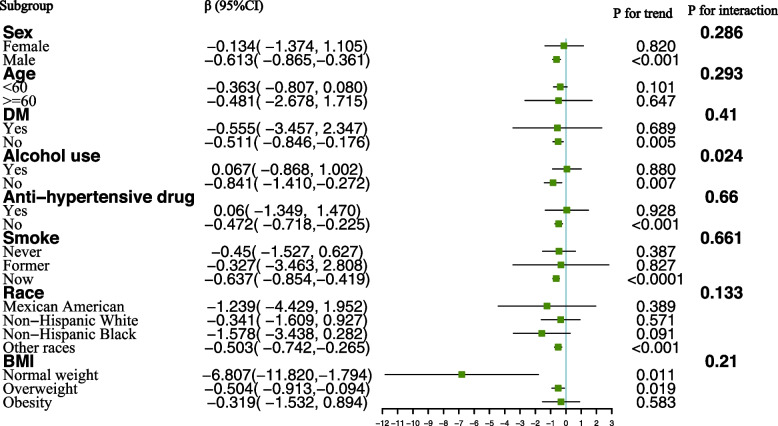
Subgroup analysis for the association between urinary chromium and diastolic blood pressure

A negative association of urinary chromium with diastolic blood pressure was observed in males (β = -0.613), participants who were current smokers (β = -0.637), overweight (β = -0.504), and other races (β = -0.503), as well as those without alcohol use (β = -0.841) and anti-hypertensive drug use (β = -0.472). The interaction term revealed the influence of alcohol consumption on the association between urinary chromium concentrations and diastolic blood pressure (P for interaction = 0.024) (Fig. 5). This suggests a significant dependence of alcohol consumption on the negative association between urinary chromium concentrations and diastolic blood pressure.

## Discussion

In this observational study that recruited 1734 subjects, we found no significant association between urinary chromium concentrations and risk of hypertension or systolic blood pressure. Besides, we also observed a negative association between urinary chromium concentrations and diastolic blood pressure. In subgroup analysis, our results suggested that urinary chromium concentrations may be associated with an increased risk of hypertension in individuals over 60 years of age. Furthermore, the negative association between urinary chromium concentrations and diastolic blood pressure appears to depend on alcohol consumption.

Chromium, an essential mineral for life, has been implicated in potentially exerting an inhibitory effect on insulin resistance. This effect may be attributed to the induction of increased levels of glucose-6-phosphatase and phosphoenolpyruvate carboxykinase 1 mRNA expression, contributing to the improvement of systemic insulin sensitivity [19]. Notably, research by Hung A. et al. suggested that chromium supplementation could enhance insulin signaling and muscle mass. This improvement might be linked to an increase in lipocalin in subcutaneous adipose tissue and a decrease in the expression of suppressor of cytokine signaling 3 (SOCS3) in skeletal muscle [20]. An animal study also found that a diet low in chromium may lead to weight gain, systemic fat accumulation, and elevated fasting triglyceride levels [21]. Basaki M et al. found significantly lower levels of serum zinc, copper, and chromium in type 2 diabetes [22]. A meta-analysis found a beneficial effect of chromium supplementation on glycemic control in diabetic patients, with both chromium monotherapy and the combination being associated with a significant reduction in HbA1c levels [23]. Additionally, this analysis reported a decrease in triglyceride levels with chromium supplementation, and importantly, it did not identify an increased risk of adverse events [23]. However, a separate study focusing on patients with polycystic ovary syndrome found no significant effect of chromium supplementation on fasting insulin and quantitative insulin sensitivity check indices [24].

Various studies have highlighted the association between urinary chromium concentrations and cardiovascular diseases, underscoring the potential impact of chromium on cardiovascular health. Chen et al. found that in male participants, low chromium levels were associated with increased odds of developing cardiovascular disease and diabetes [25]. Meng et al. identified a negative association between blood chromium levels and atherogenic cardiovascular disease [26]. Chromium concentrations in hair were found to be negatively correlated with the risk of myocardial infarction episodes. The study also noted a 24.7% decrease in chromium concentrations in the hair of individuals who succumbed to a third myocardial infarction episode compared to those who tolerated such an episode [27]. The mechanisms underlying chromium's potential reduction of cardiovascular disease risk may involve its ability to decrease the expression levels of inflammatory biomarkers associated with these risk factors. Notably, high-sensitivity C-reactive protein and tumor necrosis factor-alpha (TNF-α) have been implicated [28]. Additionally, chromium acts as an antioxidant, contributing to a reduction in malondialdehyde levels, mitigating lipid peroxidation, and regulating NF-kB activity to alleviate inflammatory responses [29, 30].

One study found that chromium supplementation significantly reduced both systolic and diastolic blood pressure, and the reduction in systolic blood pressure was greater in participants given chromium yeast [9]. An investigation into the effects of micronutrients on hypertensive patients in rural China suggested independent associations between serum concentrations of copper, selenium, and chromium with hypertension. Men with hypertension exhibited a significant decrease in serum chromium concentrations [7]. In contrast, a study involving male adolescents found no correlation between urinary chromium levels and hypertension [31].

The exact mechanism by which chromium influences blood pressure remains unclear. However, chromium supplementation has been associated with improvements in total antioxidant capacity and oxidative stress parameters, such as malondialdehyde. In animal models, chromium has been observed to facilitate insulin signaling and uphold glucose equilibrium by ameliorating endoplasmic reticulum stress [32]. Moreover, chromium demonstrates the ability to mitigate lipid accumulation through the reduction of triglyceride synthesis and the promotion of adipose tissue breakdown [33]. Additionally, chromium can impede adipogenesis by modulating the expression of the sterol regulatory element binding protein 1 (SREBP-1) gene [34]. Studies have indicated that chromium may decrease lipid peroxidation in mice by hindering carbon tetrachloride production [35]. Chromium could also reduce reactive oxygen species (ROS) and TNF-α, inhibit the expression of NF-kB, and decrease the expression of vascular cell adhesion molecule 1 (VCAM-1), thereby improving endothelial dysfunction [36]. Chromium also activates the cellular energy sensor 5'AMP-activated protein kinase (AMPK), which inhibits the activation of the NF-kB signaling pathway and the expression of inflammatory cytokines [37]. These anti-inflammatory and antioxidant properties may contribute to the regulation of blood pressure.

In hypertensive patients, significant elevations in pro-inflammatory cytokines, such as interleukin (IL)-18 and IL-1β, have been detected [38, 39]. Animal studies suggested that downregulation of MAPK and NF-kB pathways, which caused vascular inflammation, could lead to vasodilation and improve hypertension [40]. Additionally, C-reactive protein, a potent inflammatory marker associated with hypertension risk, exhibits higher levels in hypertensive and prehypertensive patients compared to those with normal blood pressure [41, 42]. In a study involving patients with coronary artery disease, chromium significantly reduced serum levels of high-sensitivity C-reactive protein [43]. Moreover, a meta-analysis revealed that chromium reduces the levels of inflammatory biomarkers such as IL-6 and TNF-α, which are major risk factors for hypertension and cardiovascular disease [44]. Chromium intake has been shown to improve blood pressure in hypertensive subjects, potentially associated with decreased renin-angiotensin system activity, reduced angiotensin-converting enzyme activity, and diminished NO activity due to inadequate bioavailability [45–48]. Additionally, chromium down-regulates the expression of Hypoxia-inducible factor 1α (HIF-1α) and up-regulates Peroxisome proliferator activated receptor α (PPARα) [49]. Activation of HIF-1α alters mitochondrial respiratory function and metabolism and affects organismal redox homeostasis, while PPARα activation is linked to the regulation of fatty acid metabolism, fatty acid oxidative catabolism, and inflammatory mechanisms [50–52]. These mechanisms related to oxidative stress and inflammation play a significant role in influencing the progression of hypertension.

The outcomes of our subgroup analyses indicate that the relationship between urinary chromium and hypertension may be influenced by racial factors. Among Non-Hispanic Black participants, we observed a positive correlation between urinary chromium and hypertension. Research proved that black adults exhibited the highest prevalence of hypertension among all racial groups in the United States [53]. Even in the pediatric population, the incidence of hypertension remains elevated in Blacks compared to Whites [54]. An examination of hypertension control rates in the U.S. populace revealed lower rates in Non-Hispanic Black individuals compared to their Non-Hispanic White counterparts [55]. Moreover, Non-Hispanic Blacks diagnosed with hypertension early in life face a substantially heightened risk of end-stage renal disease and cardiovascular death compared to Non-Hispanic Whites [56]. Racial disparities in blood pressure control may stem from lower insurance coverage and limited access to healthcare. A study uncovered significantly lower adherence to hypertension medications among patients in areas lacking routine healthcare facilities, resulting in diminished blood pressure control rates [57]. Another study highlighted that lack of insurance rates were approximately 6% higher in Non-Hispanic Blacks than in Non-Hispanic Whites [58]. Prior research has highlighted the presence of racial variations in vascular function. Healthy black women may manifest impaired microvascular function, as indicated by a diminished hemodynamic response to flow-mediated dilation compared to their healthy white counterparts [59]. Young black men exhibit greater carotid intima-media thickness, stiffer carotid arteries, reduced resistance arteriolar dilation, diminished total forearm congestive blood flow, and elevated central blood pressure in comparison to young white men [60]. A pivotal process in the progression of hypertension involves vascular inflammation, leading to the release of various pro-inflammatory cytokines that activate endothelial and vascular smooth muscle cells [61, 62]. A study identified lower blood glutathione and oxidized glutathione levels in black adults compared to their white counterparts [63]. Additionally, black adults displayed higher baseline levels of circulating C-reactive protein than whites [64]. In a study examining oxidative stress and inflammatory markers in cell culture, human umbilical vein endothelial cells from the black population demonstrated lower superoxide dismutase activity and higher levels of interleukin 6 [65]. This evidence elucidated why black people tend to exhibit greater oxidative stress and vascular inflammation.

Age also played a significant role in the correlation between urinary chromium and the risk of hypertension. Among participants aged 60 and above, higher urinary chromium concentrations were linked to an increased risk of hypertension. The prevalence of hypertension rised with age, reaching up to 74% in individuals over 80 years old [66]. Aging is intricately linked to structural and functional alterations in the arterial vascular system, encompassing both large and small arteries. For instance, aging contributes to a thickening of the arterial lining and a notable increase in the diameter of the arterial lumen in the elderly [67]. This expansion may result in repetitive stretching of elastic arteries, leading to the fatigue of elastin and eventual breakage [68, 69]. Consequently, arterial elasticity decreases, compromising the cushioning function of the arteries. This phenomenon allows the pulse wave to propagate faster, ultimately elevating systolic blood pressure levels [70]. As time progresses, pressure accumulation in the vessel wall induces overproliferation and phenotypic conversion of smooth muscle cells, culminating in the accumulation of extracellular matrix and endothelial dysfunction [71–73]. This intricate process is further associated with an imbalance in the release of vasoconstrictors and vasodilators.

The results of our subgroup analysis reveal a noteworthy negative correlation between urinary chromium concentration and diastolic blood pressure, and this correlation appears to be significantly influenced by alcohol consumption. Specifically, in non-drinkers, elevated urinary chromium concentrations are associated with a more pronounced reduction in diastolic blood pressure. Research by Roerecke M et al. indicates that any alcohol consumption increases the risk of hypertension in men. In women, the risk of hypertension is not increased at 1–2 drinks per day but is elevated with consumption exceeding 1–2 drinks [74]. Jung et al. further observed that in Asian men, even low doses of alcohol (0.01 to 20.0 g/day) led to an increased risk of hypertension. In Western men, only the high-dose alcohol group (> 60.0 g/day) showed a significantly increased risk of hypertension [75]. Chronic alcohol consumption has been associated with increased urinary levels of 20-hydroxyeicosatetraenoic acid (20-HETE), acting as a vasoconstrictor and pro-inflammatory mediator. This activation of the NF-kB pathway in endothelial cells induces the expression of the pro-inflammatory cytokine IL-8, leading to endothelial injury [76, 77]. A randomized controlled trial has also confirmed the impact of alcohol consumption on markers of endothelial function, including E-selectin and endothelin-1 [78].

There are several strengths of our study. First, this study was based on data from NHANES, a nationally representative sample of population-based data obtained through the use of a standardized protocol, and all analyses took into account appropriate NHANES sampling weights. We also adjusted for confounding covariates to ensure the robustness of the results. The cross-sectional design inherently limits the ability to establish causal relationships, highlighting the need for future large prospective cohort studies to delve deeper into causation. Despite adjusting for potential covariates, the possibility of residual confounding remains, as the effects of all potential confounders may not have been completely eliminated. Furthermore, the study's participants were drawn from one country, potentially affecting the generalizability of the findings to a global context. We did not assess whether chromium deficiency has an effect on the incidence of hypertension in this study, the development of which is determined by a combination of multiple risk factors such as dietary habits, obesity, family history, metabolic syndrome, etc., and we were unable to analyze the effect of chromium deficiency on hypertension due to the lack of data on daily dietary chromium intake in the NHANES database. This consideration emphasizes the importance of cautious interpretation and encourages future research to validate and expand upon these findings. Last but not least, despite its limitations, NHANES has become a valuable resource for longitudinal assessment of the clinical epidemiology of hypertension in the US population.

## Conclusion

In our current study, we observed no significant correlation between urinary chromium concentration and hypertension or systolic blood pressure. However, a notable negative correlation was identified between urinary chromium concentration and diastolic blood pressure. Subgroup analysis results indicated a potential association between urinary chromium concentration and an elevated risk of hypertension in individuals aged over 60 and those who were Non-Hispanic Black. Furthermore, the negative correlation between urinary chromium concentration and diastolic blood pressure appeared to be influenced by alcohol consumption. It is important to emphasize that while these findings provide valuable insights, further validation is warranted through large-scale prospective studies.

## Data Availability

The datasets generated and/or analysed during the current study are available in the NHANES repository, https://www.cdc.gov/nchs/nhanes/index.htm.
